# *BDH1* identified by transcriptome has a negative effect on lipid metabolism in mammary epithelial cells of dairy goats

**DOI:** 10.1186/s12864-025-11245-1

**Published:** 2025-01-24

**Authors:** Mengke Ni, Saige Zang, Yihan Wang, Xiaochen Qin, Min Tian, Tiantian Xiong, Chong Chen, Yue Zhang, Jun Luo, Cong Li

**Affiliations:** College of Animal Science and Technology, Northwest A&F University, Yangling, 712100 China

**Keywords:** *BDH1*, Mammary epithelial cells, Lipid metabolism, Expression regulation, Dairy goats

## Abstract

**Background:**

The 3-hydroxybutyrate dehydrogenase 1 (*BDH1*) mainly participates in the regulation of milk fat synthesis and ketone body synthesis in mammary epithelial cells. In our previous study, *BDH1* was identified as a key candidate gene regulating lipid metabolism in mammary glands of dairy goats by RNA-seq. This study aimed to investigate the effect of *BDH1* on lipid metabolism in mammary epithelial cells of dairy goats (GMECs).

**Results:**

The results suggest that *BDH1* plays a significant role in reducing triacylglycerol content and lipid droplet accumulation in GMECs (*p* < 0.05). Overexpression of *BDH1* significantly decreased the expression of lipid metabolism-related genes (*SREBF1* and *GPAM*) and reduced the levels of C14:0 and C17:1, while increasing *FABP3* expression and C10:0 concentration (*p* < 0.05). Interference with *BDH1* significantly increased the expression of *SREBF1* and *GPAM* and the concentration of C14:0, C15:1, and C20:1, but significantly decreased *FABP3* and C18:0 (*p* < 0.05). Treatment of GMECs with β-hydroxybutyric acid (R-BHBA) significantly decreased the expression of *FASN*, *ACACA*, *LPL*, *SREBF1*, *FABP3*, *ACSL1*, *GPAM*, *DGAT1*, and triacylglycerol content, while significantly increasing the expression of *BDH1* (*p* < 0.05). Interference with *BDH1* rescued the reduction of cellular TAG content and the expression of *FASN*, *LPL*, *SREBF1*, *ACSL1*, and *GPAM* in BHBA-treated GMECs.

**Conclusion:**

In conclusion, *BDH1* negatively regulates lipid metabolism in mammary glands of dairy goats. Furthermore, it may mitigate the inhibitory effect of R-BHBA on lipid metabolism in GMECs.

**Graphical Abstract:**

BDH1 serves as a negative regulator of milk lipid synthesis in GMECs, and BDH1 counteracts the inhibitory effect of R-BHBA on lipid synthesis in mammary epithelial cells of dairy goats.

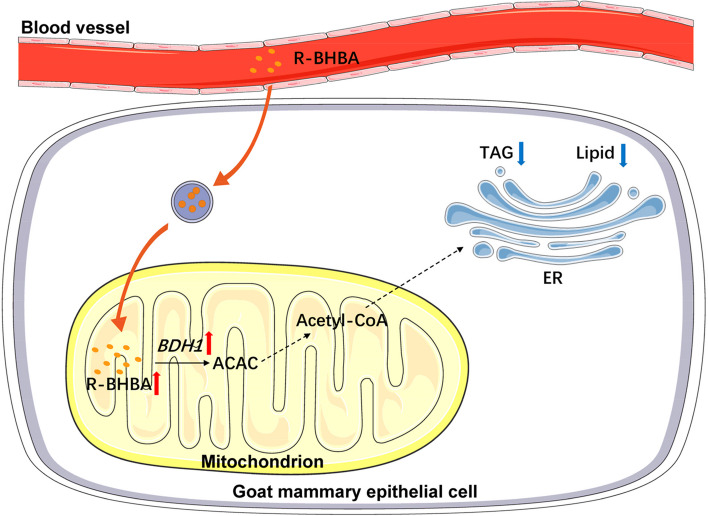

**Supplementary Information:**

The online version contains supplementary material available at 10.1186/s12864-025-11245-1.

## Highlights

• BDH1 has a negative regulatory effect on lipid metabolism in GMECs.

• R-BHBA negatively regulated milk fat metabolism and TAG synthesis in GMECs.

• R-BHBA and BDH1 positively regulated the milk lipid metabolism of dairy goats.

**Supplementary Information:**

The online version contains supplementary material available at 10.1186/s12864-025-11245-1.

## Background

Goat milk is recognized as an essential component of human nutrition due to its superior composition. Notably, goat milk contains higher protein, fat and mineral contents with a nutritional profile that closely aligns with human milk [1]. Goat milk has a higher proportion of small milk fat globules (each less than 3.5 μm in diameter), making the fats and proteins in goat milk more easily digestible and absorbable [2]. Moreover, goat milk is rich in short-chain fatty acids and monounsaturated fatty acids. Approximately 32.42% of total fatty acids in goat milk are composed of C6 to C14 fatty acids [3]. Additionally, goat milk contains higher levels of long-chain unsaturated fatty acids, including linoleic acid, linolenic acid, and neuronic acid [4]. Beyond its nutritional richness, goat milk has been associated with health benefits, contributing to the prevention of metabolic diseases such as hyperlipidemia, nutrient absorption disorders, nutritional deficiencies, and diabetes [5–7]. Therefore, goat milk is considered a natural source of nutrients with higher nutritional value.

The fatty acids required for milk fat synthesis in the mammary gland of ruminants originate from two sources, direct uptake from the blood and de novo synthesis in mammary epithelial cells [8]. Among the main raw materials for the de novo synthesis of fatty acids are acetic acid (HAc) and β-hydroxybutyric acid (BHBA). Ketone bodies, produced during fat oxidation and catabolism, include acetoacetic acid (ACAC), β-hydroxybutyric acid (BHBA), and acetone. Ruminants, particularly in the lactating mammary gland, metabolize significant amounts of BHBA [9, 10]. Previous studies have highlighted the role of 3-hydroxybutyrate dehydrogenase 1 (*BDH1*) in regulating milk fat synthesis in buffalo mammary epithelial cells and ketone body synthesis and utilization in mouse hepatocyte [11–13]. The *BDH1* facilitates the interconversion of acetoacetate and (R)-3-hydroxybutyrate, particularly promoting the utilization of BHBA [14]. In buffalo mammary epithelial cells, the expression pattern of *BDH1* follows the lactation curve. Expression levels increase as lactation progresses, peaking at 60 days, and then decrease as lactation nears its end. Throughout lactation, *BDH1* maintains consistently high expression, demonstrating a strong negative correlation between *BDH1* expression pattern and milk fat yield [11].

In our previous study, we conducted RNA-seq on the mammary tissues of dairy goats with low (< 3%) and high (> 4.5%) milk fat percentages. The results indicated that *BDH1* expression (log_2_[Fold Change] = -0.69, *p* = 0.004) was significantly lower in the high milk fat percentage group. Therefore, we hypothesized that *BDH1* is a key candidate gene for regulating lipid metabolism in the mammary glands of dairy goats. However, there is limited research on the role of *BDH1* in lipid metabolism of dairy goats. Therefore, this study employed overexpression and RNA interference techniques to investigate the role and mechanism of *BDH1* in lipid metabolism in GMECs. This research provides a theoretical foundation for further in-depth exploration of the regulatory mechanisms of *BDH1* in lipid metabolism in GMECs.

## Materials and methods

### Ethics approval and consent to participate

All the experimental procedures were performed under the approval of the Institutional Animals Care and Use Committee (IACUC) of Northwest A&F University, China (Approval No. DK2021054).

### Transcriptome sequencing and data analysis

The RNA-seq library was prepared by using the TruSeq RNA Sample Preparation Kit (Illumina, USA). The raw data (short reads) obtained from transcriptional sequencing of liver tissue were processed using FASTP (version 0.21.1) with default parameters. The index of ARS1.2 was generated through the HISAT2 (version 2.2.1) build software. Subsequently, clean data were aligned to ARS1.2 using HISAT2. Transcript abundance and gene abundance were evaluated based on the reads per million mapped reads (FPKM) and per kilobase fragment. De novo assembly and annotation identified the differentially expressed genes (DEGs) between two groups. Volcano map was plotted with ggplot2 in R (version 3.4.2).

### Bioinformatics analysis of *BDH1*

The amino acid sequence of *BDH1*, along with the routine physicochemical properties of the protein and its signal peptide, were analyzed using DNAMAN 8.0 software, Protparam online software and TargetP 1.1 Server software, respectively (Table S1). The secondary and tertiary structures of BDH1 protein were predicted using NPS online software and Phyre2 website (version 2.2), respectively. Phosphorylation sites were predicted by NetPhosK (version 3.1) and the transmembrane structural domains were analyzed by TMHMM (version 2.0) of the *BDH1*. The protein–protein interaction networks (PPI) of BDH1 proteins were predicted using the STRING online database. Homology between species was analyzed by BLAST sequence alignment, and the evolutionary tree was constructed using MEGA 6.0.

### Cell culture and BHBA treatment

The GMECs from healthy lactating dairy goats used in this study were obtained from our previous research, where they were isolated and purified to ensure high cell viability and lactation potential [15]. Briefly, mammary tissue pieces (0.5 to 1 mm^3^) were transferred into 24-well cell culture plate and cultured in growth medium [DMEM/F12 supplemented with 10% fetal bovine serum (FBS, Gibco, USA), 10 ng/mL epidermal growth factor (EGF, PHG0313, Gbico, USA), 5 μg/mL insulin (MC0669, NOVON, China), 5 mg/L hydrocortisone (088–02483, MP BIOMEDICALS, China), and 1% penicillin–streptomycin (Pricella, China)] at 37 °C and 5% CO_2_. The medium was replaced every 48 h until the cells migrated out of the tissue. The isolated cells attached to the dish surface were passaged by digestion with 0.25% trypsin/ethylenediaminetetraacetic acid (EDTA). Pure GMECs were selected after 2 passages and used between passages 3 and 7 for the experiments.

Before 12 h of treatment, cells were switched to serum-free medium, where fetal bovine serum (FBS) was replaced with fatty acid-free bovine serum albumin (BSA) (1 g/L, Solarbio, China). GMECs were treated with (R)-β-hydroxybutyric acid at concentrations of 0, 0.8, 1.6, 2.4, 3.2, and 4.0 mmol/L for 24 and 48 h, respectively.

### Cell transfection

Based on the predicted sequence of goat *BDH1* from NCBI (XM_018046468.1), specific cloning primers and enzyme digestion primers (Hind III, Xba I) for *BDH1* were designed using Primer Premier 6.0 software and Primer-BLAST on NCBI. Additionally, small interfering RNA (siRNA) was designed based on the CDS sequence of *BDH1*. The primer sequences are provided in Table S2 and S3. The cDNA of *BDH1* was amplified and cloned into pcDNA3.1 vector. All expression constructs were verified by Sanger sequencing. *BDH1* plasmids were used at 50 nmol/L for transient transfection. Overexpression and interference experiments were conducted using Lipofectamine 2000 (Invitrogen) and Lipofectamine RNAiMAX Reagent (Invitrogen), respectively.

### RNA extraction and quantitative Real Time PCR (RT-qPCR)

Total RNA from cells was extracted using TRIzol reagent (Invitrogen, CA, USA) according to the manufacturer’s instructions. The concentration and integrity of the total RNA were measured using the NanoDrop One spectrophotometer (Thermo Scientific, USA) and agarose gel electrophoresis. First-strand cDNA was synthesized using a PrimeScript RT kit (TaKaRa, Japan). RT-qPCR assays were performed using SYBR Premix Ex Taq II kit (TaKaRa, Japan) to determine mRNA expression of genes on a QuantStudio 5 (Applied Biosystems, USA), following the previously published protocols [16]. Gene specific primers (Table S4) were designed and synthesized by Sangon Biotech (China) and were optimized before the initial screening and quantitative analysis.

### Western blot

Total protein from cells was lysed with RIPA lysis buffer (PC101, Beyotime, China) supplemented with phenylmethanesulfonyl fluoride (PMSF, Epizyme Biotech, China) and a phosphatase inhibitor cocktail (Epizyme Biotech, China). Protein concentration was determined using the BCA kit (Epizyme Biotech, China). A 10% SDS-PAGE gel was used to separate proteins, followed by transfer to a polyvinylidene difluoride membrane. The membrane was then blocked with 5% BSA for 1 h at room temperature and subsequently incubated with primary antibodies at 4 °C overnight. The primary and secondary antibodies used in this study are shown in Table S5. Afterward, the member was incubated with a secondary antibody for 1 h at room temperature. Blots were visualized using the Omni-ECL Femto Light Chemiluminescence Kit (Epizyme Biotech, China) on a QuickChemi 5200 chemiluminescence imaging system (Monad Biotech, China). All protein levels were analyzed with Image J and normalized to β-Tubulin.

### BODIPY staining

The BODIPY staining was performed according to the manufacturer’s instructions. Briefly, cells were fixed for 30 min at 4 °C using 4% Paraformaldehyde. After discarding the paraformaldehyde, 300 µL of BODIPY staining solution (C2053S, Beyotime) was added to each well under light-avoidance conditions for an additional 30 min. Subsequently, 200 µL of DAPI nuclear staining solution (C1002, Beyotime) was added.

### Triacylglycerol assay

The triacylglycerol (TAG) content in GMECs was determined using assay kits from Nanjing Jiancheng Bioengineering Institute according to the manufacturer’s procedures (Jiancheng, China). The results were detected using a microplate reader (Synergy LX, BioTek, USA).

### Fatty acid profile

Fatty acid composition and content of GMECs were analyzed by GC–MS. Briefly, GMECs were homogenised with chloroform/methanol (2:1, v/v) and mixed with brine solution (0.7% KCl). The aqueous phase was discarded, and the lipid-chloroform fraction was evaporated using a rotary evaporator. Subsequently, 4.0 g of anhydrous sodium sulphate was added, shaken, and left to stand for 5 min. The upper layer of the solution was pipetted through an organofiltration membrane and then diluted 1000-fold for the subsequent gas chromatography analysis. The internal standard method was used with an Agilent 7890A gas chromatograph coupled with an Agilent 5975C quadrupole mass spectrometer [17].

### Cell Counting Kit-8 (CCK-8) assay

The GMECs seeded in 96-well plates (Corning, USA) were incubated with different concentrations (0, 0.8, 1.6, 2.4, 3.2, 4.0 mmol/L) of BHBA for different time durations (24 h, 48 h). After incubation, the plates were further incubated for 2 h with 10 µL of CCK-8 (CK04, Dojindo, Japan) per well. Absorbance was measured at 450 nm using a microplate reader (Synergy LX, BioTek, USA) for 96-well plate readings.

### Statistical analysis

All data are presented as the means ± SEM of at least three independent experiments. Statistical significance was determined with two-tailed Student’s t-tests or ANOVA, as indicated in the figure legends, with statistical package for the social sciences (SPSS, USA) and Prism 9 (GraphPad, USA) software. Relative gene expression levels were normalized to the the endogenous RNA control *RPS9* and *UXT* with the 2^−ΔΔCq^ method. Densitometric quantification of western blot bands was performed using Image J (National Institutes of Health, USA). All statistical analyses were defined as highly significant at *P* < 0.01, significant at *P* < 0.05 and a trend at 0.05 < *P* < 0.10.

## Results

### Strategy for identification of key candidate gene *BDH1*

In our previous study, the whole transcriptome was performed on mammary tissues of dairy goats with low and high milk fat percentages. The results indicated that *BDH1* expression (log_2_[Fold Change] = -0.69, *p* = 0.004) was significantly lower in the high milk fat percentage group. (Table S6). The distribution of differentially expressed genes (DEGs) is illustrated in a volcano plot, with the location of *BDH1* highlighted in Fig. S1.

### Cloning, sequence analysis and tissue expression of goat *BDH1*

**Fig. 1:**
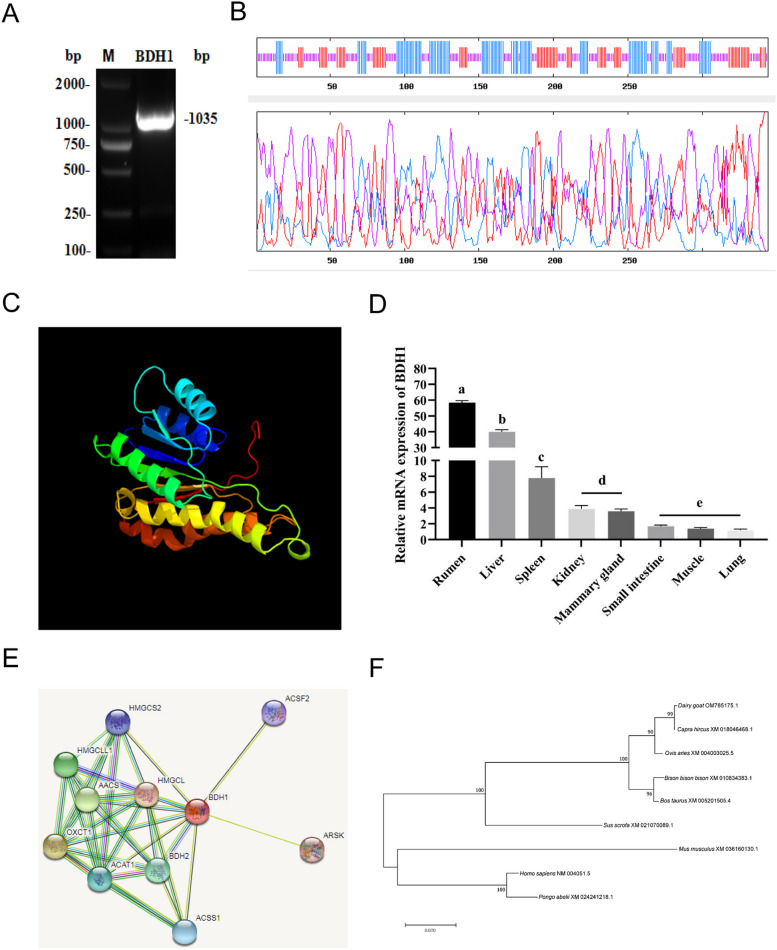
Cloning, sequence analysis and tissue expression of goat *BDH1*. **A** Cloning of *BDH1* in goat. M: DL2000 DNA Marker. **B** Prediction of secondary structure of *BDH1* protein in dairy goat. **C** Prediction of tertiary structure of *BDH1* protein in dairy goat. **D** Tissue expression profiles of *BDH1* in dairy goats. **E** Protein–protein interaction networks of *BDH1*. **F** Evolutionary tree for amino acid sequences of *BDH1* in dairy goat. ^*^p < 0.05, ^**^p < 0.01, ^***^p < 0.001. Values are represented as means ± SEM (*n* = 3). The same as below

To further investigate the function of the BDH1 gene, we initially conducted bioinformatics and differential tissue expression analyses on its CDS region sequence. PCR was used to amplify the 1,035 bp coding sequence (CDS) region of *BDH1*, using GMECs cDNA as a cloning template (GenBank: OM785175.1). The region was identified by plasmid biallelic identification and positive plasmid sequencing for sequence comparison (Fig. 1A). Analysis of the secondary structure of BDH1 protein revealed that three forms were present in the secondary structure of BDH1: 44.48% randon coil, 30.52% α-helical and 25% β-folded (Fig. 1B). Additionally, the tertiary structure of BDH1 protein mirrored the secondary structure (Fig. 1C).

Normalized to the expression in the small intestine, *BDH1* showed the highest expression in the rumen of dairy goats, and medium level expression in mammary glands (Fig. 1D). Protein–protein interaction network prediction from the STRING database showed that BDH1 may interact with BDH2, HMGCL, ACAT1, OXCT1, ACSS1, HMGCLL1, AACS, HMGCS2, ARSK, and ACSF2 (Fig. 1E). Comparisons of the *BDH1* CDS sequence of dairy goats with *Capra hicrus* (XM_018046468.1), *Ovis aries* (XM_004003025.4), *Bos taurus* (XM_005201505.4), *Bison bison* (XM_010834383.1), and *Mus musculus* (XM_036160130.1) by BLAST on NCBI revealed high homology with goat (100%), sheep (99.60%) and cattle (99.01%). The closest relationships were observed with goat, sheep, and cattle, while the more distant relationships were with human and mice (Fig. 1F).

The amino acid sequence of BDH1 contains 37 negatively charged residues and 42 positively charged residues, suggesting BDH1 may be a positively charged basic protein (Fig. S2). There are 37 phosphorylation sites, including 23 serine (Ser), 8 threonine (Thr) and 6 tyrosine (Tyr) phosphorylation sites (Fig. S3). Moreover, BDH1 lacks a transmembrane structural domain (Fig. S4).

### *BDH1* promotes lipid metabolism and affects fatty acid synthesis in GMECs

**Fig. 2:**
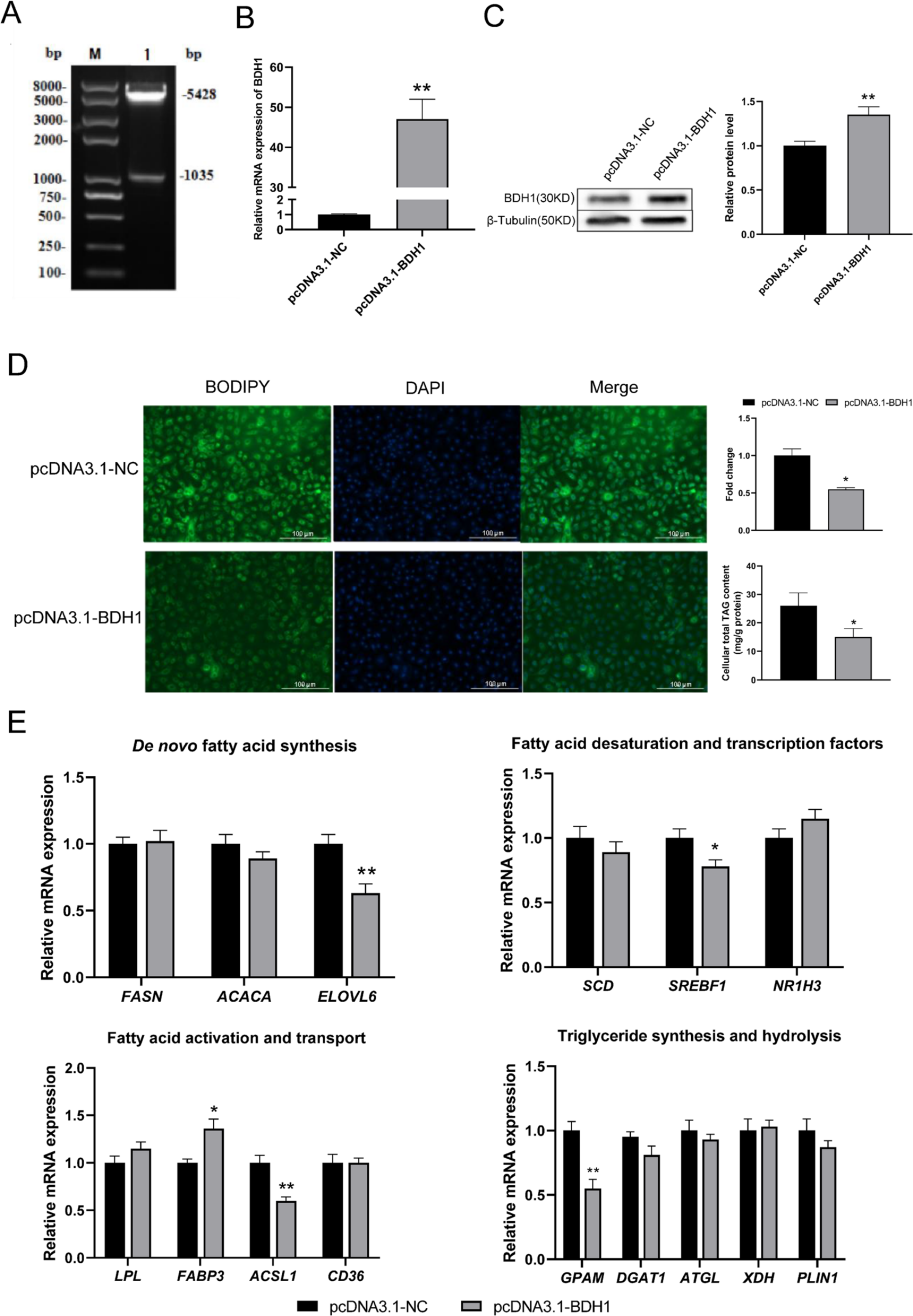
Regulation of *BDH1* overexpression on lipid metabolism in GMECs. **A** Results of double-enzyme digestion of pcDNA3.1-BDH1. Changes in (**B**) relative mRNA expression and (**C**) protein level of *BDH1*. **D** Effects of overexpression of *BDH1* on cellular lipid droplet accumulation and total TAG content. **E**–**F** Overexpression of *BDH1* affects the relative mRNA expression of genes related to milk fat synthesis

To construct the overexpression plasmid for the *BDH1*, pcDNA3.1-*BDH1* was identified by double enzyme digestion with Q.Cut Hind III and Q.Cut XbaI, resulting in band sizes of 5,428 bp and 1,035 bp, respectively (Fig. 2A). To explore the regulatory mechanism of *BDH1* on lipid metabolism, pcDNA3.1-NC and pcDNA3.1-*BDH1* plasmids were transferred into GMECs for 48 h. Total RNA and total protein were then extracted. Relative mRNA expression levels of *BDH1* increased approximately 50-fold (Fig. 2B), and BDH1 protein levels were significantly elevated compared to control group (Fig. 2C).

The BODIPY staining assay results revealed that overexpression of *BDH1* significantly decreased the accumulation of intracellular lipid droplets. Additionally, the intracellular TAG content of GMECs was significantly decreased (*p* < 0.05) (Fig. 2D). By detecting the effect of overexpression of *BDH1* on genes related to lipid metabolism in GMECs, we observed a significant decrease in the relative mRNA expression of *ELOVL6*, *SREBF1*, *ACSL1*, and *GPAM*, and a significant increase in *FABP3* (*p* < 0.05) (Fig. 2E).

The intracellular fatty acid content of GMECs showed that overexpression of *BDH1* significantly up-regulated the amount of C10:0 and significantly down-regulated the amount of C14:0 and C17:1 (*p* < 0.05). However, there was no significant effect on C16:0 and C18:0 (Table 1).

**Table 1:** Effects of overexpression of *BDH1* on fatty acid composition in GMECs

Fatty acid (%)	pcDNA3.1-NC	pcDNA3.1-BDH1	*p*-Value
C10:0	0.32 ± 0.05^B^	0.81 ± 0.07^A^	0.005
C12:0	0.30 ± 0.08	0.29 ± 0.10	0.074
C14:0	4.48 ± 0.22^a^	3.48 ± 0.15^b^	0.012
C14:1	2.29 ± 0.32	1.96 ± 0.23	0.442
C15:0	0.45 ± 0.04	0.35 ± 0.01	0.107
C16:0	47.47 ± 0.53	48.32 ± 0.29	0.236
C16:1	0.90 ± 0.08	0.69 ± 0.06	0.174
C17:0	0.79 ± 0.02	0.86 ± 0.11	0.210
C17:1	1.24 ± 0.10^A^	0.63 ± 0.08^B^	0.009
C18:0	21.35 ± 0.05	21.14 ± 0.20	0.353
C18:1	12.76 ± 1.01	13.01 ± 0.60	0.851
C18:2	3.25 ± 0.11	2.89 ± 0.23	0.089
C20:1	0.34 ± 0.02	0.32 ± 0.02	0.476
C20:3	0.27 ± 0.01	0.26 ± 0.02	0.585
C20:4	0.63 ± 0.08	0.61 ± 0.04	0.844
C20:5	0.25 ± 0.03	0.24 ± 0.01	0.602
C21:0	0.07 ± 0.00	0.08 ± 0.01	0.617
C22:6	0.49 ± 0.04	0.52 ± 0.04	0.668

^A,B^Capital letters indicate *p* < 0.01

^a,b^Lowercase letters indicate *p* < 0.05. Values are represented as means ± SEM (*n* = 3)

### Interference with *BDH1* promotes lipid metabolism and affects fatty acid synthesis in GMECs

**Fig. 3:**
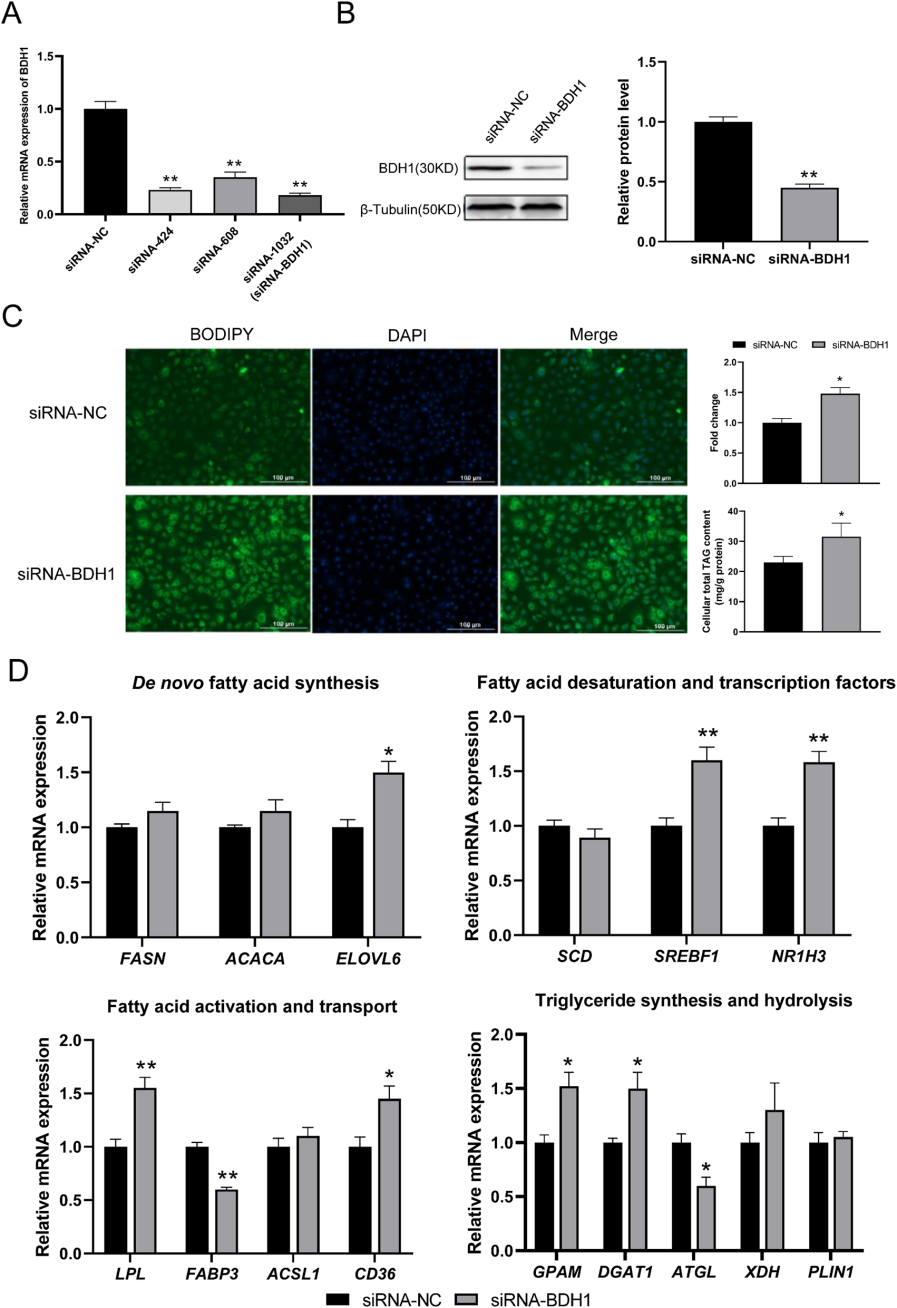
Regulation of *BDH1* interference on lipid metabolism in GMECs. Changes in (**A**) relative mRNA expression and (**B**) protein level of *BDH1*. Effects of interference of *BDH1* on (**C**) cellular lipid droplet accumulation and total TAG content. **D**-**G** Interference of *BDH1* affects the relative mRNA expression of genes related to milk fat synthesis

To explore the regulatory mechanism of *BDH1* on lipid metabolism, three siRNAs were designed and individually transfected into GMECs for 48 h. siRNA-1032 (named siRNA-BDH1) was selected as the most efficient for subsequent experiments based on the results (Fig. 3A). Meanwhile, the protein level of *BDH1* significantly decreased, consistent with the trend of mRNA expression (*p* < 0.05) (Fig. 3B).

To investigate the effects of *BDH1* on intracellular lipid droplet accumulation in GMECs, we used the BODIPY staining assay to observe lipid droplets. The results revealed that the interference of *BDH1* significantly increased the accumulation of intracellular lipid droplets (*p* < 0.05). Additionally, the intracellular TAG content in GMECs was significantly increased (*p* < 0.05) (Fig. 3C). By detecting the effect of interfering *BDH1* on genes related to lipid metabolism in GMECs, we observed that the relative mRNA expression of *ELOVL6*, *SREBF1*, *NR1H3*, *LPL*, *CD36*, *GPAM*, and *DGAT1* were significantly increased, while *FABP3* and *ATGL* was significantly decreased (*p* < 0.05) (Fig. 3D).

The intracellular fatty acid content of GMECs revealed that interference with *BDH1* significantly up-regulated the amount of C14:0, C14:1, C15:1, C20:1, and C20:4 and significantly down-regulated the amount of C18:0 (*p* < 0.05) (Table 2).

**Table 2:** Effects of interference with *BDH1* on fatty acid composition in GMECs

Fatty acid (%)	siRNA-NC	siRNA-BDH1	*p*-Value
C10:0	0.23 ± 0.06	0.48 ± 0.22	0.264
C12:0	0.17 ± 0.03	0.40 ± 0.12	0.248
C14:0	4.46 ± 0.26^b^	6.92 ± 0.79^a^	0.036
C14:1	1.90 ± 0.11^B^	3.47 ± 0.26^A^	0.007
C15:0	0.40 ± 0.00	0.41 ± 0.09	0.953
C15:1	0.68 ± 0.01^B^	0.85 ± 0.00^A^	0.003
C16:0	44.42 ± 1.96	37.45 ± 5.82	0.320
C16:1	0.70 ± 0.07	0.47 ± 0.24	0.411
C17:0	0.72 ± 0.03	0.72 ± 0.03	0.980
C17:1	0.82 ± 0.05	1.10 ± 0.08	0.107
C18:0	20.68 ± 0.13^A^	18.44 ± 0.12^B^	0.006
C18:1	12.77 ± 0.24	11.89 ± 0.38	0.122
C18:2	3.76 ± 0.01	3.75 ± 0.03	0.700
C20:1	0.54 ± 0.00^b^	0.62 ± 0.02^a^	0.020
C20:3	0.44 ± 0.01	0.41 ± 0.06	0.744
C20:4	1.05 ± 0.03^b^	1.20 ± 0.03^a^	0.047
C20:5	0.39 ± 0.01	0.44 ± 0.02	0.053
C20:6	0.94 ± 0.03	0.98 ± 0.14	0.809

^A,B^Capital letters indicate *p* < 0.01

^a,b^Lowercase letters indicate *p* < 0.05. Values are represented as means ± SEM (*n* = 3)

### Effect of BHBA on lipid metabolism in GMECs

**Fig. 4:**
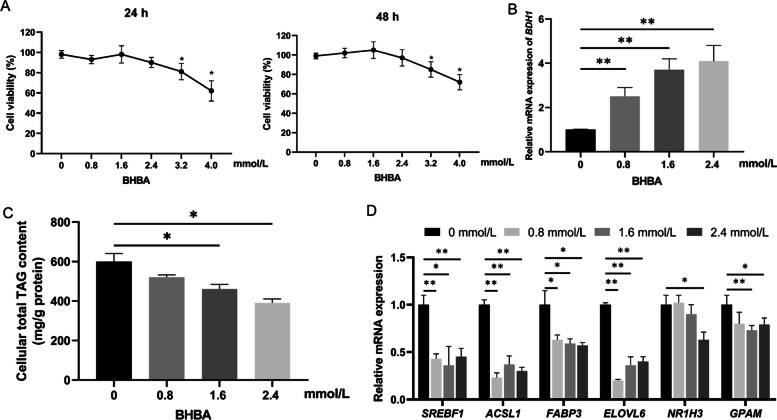
Effect of BHBA on lipid metabolism in GMECs. **A** Effect of different concentrations of BHBA treatment for 24 h and 48 h on cell activity. **B** Effects of BHBA with different concentrations on the relative mRNA expression of *BDH1*. The statistical analysis in (**A**) and (**B**) was compared with the control group. **C** Effects of BHBA with different concentrations on cellular total TAG content. **D** Effects of BHBA with different concentrations on the relative mRNA expression of lipid metabolism-related genes

The CCK-8 assay was used to determine the most appropriate concentration of BHBA to add to GMECs. A BHBA concentration of 1.6 mmol/L was the highest concentration that did not affect cell viability after 24 h and 48 h of treatment (Fig. 4A). With the increasing concentration of BHBA treatment, the relative mRNA expression level of *BDH1* significantly increased in a dose-dependent manner (*p* < 0.05) (Fig. 4B). Concentrations with 1.6 mmol/L and 2.4 mmol/L of BHBA significantly decreased the intracellular TAG content in GMECs for 48 h (*p* < 0.05) (Fig. 4C).

To investigate the effects of BHBA on lipid metabolism-related genes in GMECs, cells were cultured with starvation medium containing fatty acid-free BSA for 12 h when the cell density was about 70%, and then treated with BHBA at concentrations of 0, 0.8, 1.6 and 2.4 mmol/L to 24 h, respectively. When cells were treated with 0.8 mmol/L BHBA, the relative mRNA expression of *SREBF1*, *ACSL1*, *FABP3*, and *ELOVL6* was significantly decreased (*p* < 0.05). The relative mRNA expression of *SREBF1*, *ACSL1*, *FABP3*, *ELOVL6*, and *GPAM* was significantly decreased when the cells were treated with 1.6 mmol/L BHBA (*p* < 0.05). The relative mRNA expression of *SREBF1*, *ACSL1*, *ELOVL6*, *FABP3*, *NR1H3* and *GPAM* was significantly decreased when the cells were treated with 2.4 mmol/L BHBA (*p* < 0.05) (Fig. 4D). Considering the effects of BHBA on cell viability, lipid metabolism-related genes, and triglyceride levels, the final treatment concentration of BHBA was determined to be 1.6 mmol/L.

### Effect of *BDH1* on lipid metabolism-related genes in GMECs treated with BHBA

**Fig. 5:**
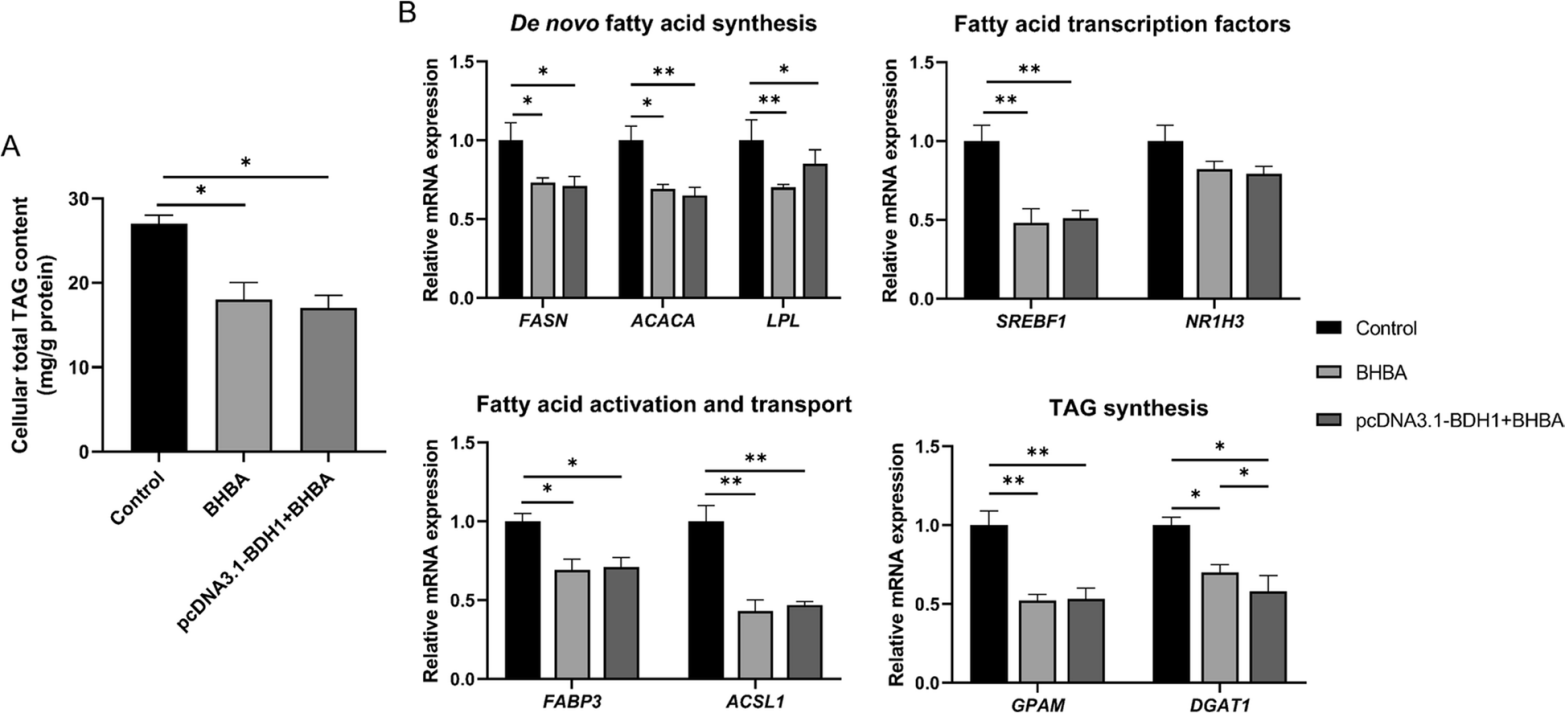
Effect of *BDH1* on lipid metabolism-related genes in BHBA-treated GMECs. **A** The effect of overexpression of *BDH1* on cellular total TAG content in BHBA-treated GMECs. **B** Effects of overexpression of *BDH1* on genes related to lipid metabolism in BHBA-treated GMECs. Control = pcDNA3.1-NC + ddH_2_O, BHBA = pcDNA3.1-NC + BHBA

The TAG content in GMECs co-treated with pcDNA3.1-BDH1 and BHBA did not significantly decrease compared to the group treated with BHBA alone (Fig. 5A). Probably because the level of fatty acids in GMECs has dropped to a threshold. The relative mRNA expression of *FASN*, *ACACA*, *LPL*, *SREBF1*, *FABP3*, *ACSL1*, *GPAM*, and *DGAT1* genes significantly decreased in GMECs treated with BHBA alone and in those co-treated with pcDNA3.1-BDH1 and BHBA (*p* < 0.05). Compared with GMECs treated with BHBA-only, the relative mRNA expression of *DGAT1* was significantly decreased in the co-treatment of pcDNA3.1-*BDH1* with BHBA (*p* < 0.05) (Fig. 5B).

### Effects of interference with *BDH1* on lipid metabolism-related genes in BHBA-treated GMECs

**Fig. 6:**
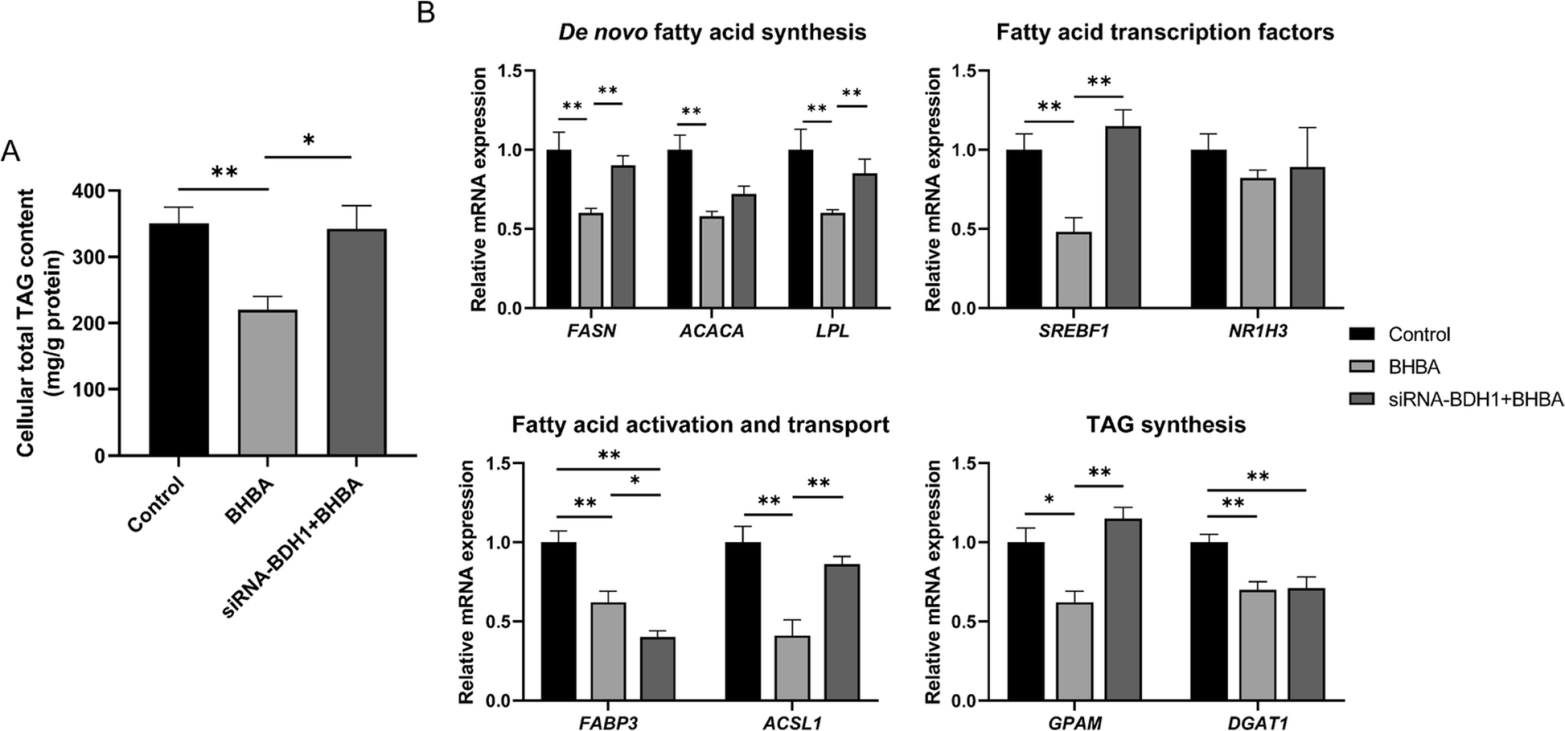
Effects of interference with *BDH1* on lipid metabolism-related genes in BHBA-treated GMECs. **A** The effect of interfering *BDH1* on cellular total TAG content in BHBA-treated GMECs. **B** Effects of *BDH1* interference on genes related to lipid metabolism in BHBA-treated GMECs. Control = siRNA-NC + ddH_2_O, BHBA = siRNA-NC + BHBA

Interference with *BDH1* expression rescued the reduction of TAG content in BHBA-treated GMECs (*p* < 0.05) (Fig. 6A). The relative mRNA expression of *FASN*, *ACACA*, *LPL*, *SREBF1*, *FABP3*, *ACSL1*, *GPAM* and *DGAT1* were significantly decreased in GMECs treated with BHBA (*p* < 0.05). Compared with GMECs treated with BHBA alone, co-treatment of siRNA-BDH1 with BHBA significantly increased the relative mRNA expression of *FASN*, *LPL*, *SREBF1*, *ACSL1*, and *GPAM*, but decreased the expression of *FABP3* (*p* < 0.05).

## Discussion

In 1992, the *BDH1* was first cloned in rat liver and human heart and subsequently reported in multiple species [18, 19]. To date, no research has been published on the *BDH1* sequence in goats. Therefore, in this study, the *BDH1* sequences of goats, cows, human, mice, and pigs were compared using BLAST. Subsequently, the *BDH1* sequence of the goat with the highest homology and closest relationship was selected as the reference, and specific PCR cloning primers were designed for this reference sequence.

During the early stages of lactation in dairy animals, *VLDLR* and *LPL* collaborate to regulate the hydrolysis of blood TAG and facilitate the release of LCFA from VLDL. Mammary epithelial cells actively uptake LCFA and stimulate fatty acid metabolism through CD36. De novo fatty acid synthesis is primarily mediated by FASN and ACACA. Partial fatty acids are desaturated by *SCD* in the product of de novo synthesis and are catalytically processed to TAG by *DGAT1*. *GPAM*, *LPIN1*, and *AGPAT6* within mitochondria are also crucial in the fatty acid modification process mentioned above [20].

The BDH1 protein–protein interaction network was predicted by the STRING database, revealing interactions with lipid metabolism-related genes, such as acetyl-CoA acetyltransferase 1 (ACAT1), acyl-CoA synthetase short chain family member 1 (ACSS1) and acetoacetyl-CoA synthetase (AACS). ACAT1 encodes an enzyme synthesizing cholesterol esters in cells, contributing to cellular cholesterol homeostasis [21, 22]. Additionally, ACAT1 plays a role in the oxidative decomposition of fatty acids to acetyl-CoA, the final step in mitochondrial β oxidation [23]. ACSS1 encodes the enzyme catalyzing the synthesis of acetyl-CoA from short-chain fatty acids and has been identified as a fat-related enzyme in mammary epithelial cells of dairy cow [24, 25]. AACS activates acetoacetic acid to acetyl acetyl coenzyme A, which may be able to use ketone bodies for fatty acid synthesis during adipose tissue development [26]. These findings suggest that *BDH1* may have a regulatory effect on lipid metabolism in dairy goats.

In studies on lactating cows, yaks, and buffaloes, *BDH1* has been identified as a crucial candidate gene for regulating lipid metabolism [11, 12, 27]. In this study, overexpression and interference methods were employed to investigate the effect of *BDH1* on lipid metabolism related genes in dairy goats. The RT-qPCR results demonstrated that overexpression of *BDH1* inhibited milk fat synthesis in GMECs, potentially through the regulation of *SREBF1*, *ELOVL6*, *ACSL1*, *GPAM*, and *FABP3*. Meanwhile, interference with *BDH1* promoted milk fat synthesis in GMECs, possibly by regulating *SREBF1*, *NR1H3*, *LPL*, *ELOVL6*, *CD36*, *GPAM*, *DGAT1*, *FABP3* and *ATGL*. Therefore, *BDH1* likely inhibits lipid metabolism in GMECs, acting synergistically with *SREBF1*, *ELOVL6*, and *GPAM*, and antagonistically with *FABP3* in this process.

Yadav et al. reported a negative correlation between *BDH1* and *SREBF1* in regulating lipids in mammary epithelial cells of buffaloes [11]. Findings by Xu et al. indicate that the *SREBF1* gene directly regulates the *ELOVL6* gene through the SRE locus, which in turn affects the change of fatty acid composition [28]. *FABP3*, a member of the fatty acid binding protein family, is widely distributed in tissues with a high demand for fatty acids, such as myocardium, skeletal muscle, and lactating breast [29]. *FABP3* is involved in the intracellular transport and transient storage of fatty acids, facilitating their entry into the mitochondrial energy metabolism system for oxidative catabolism and ATP production, thereby supplying energy to tissues [30]. The *GPAM* catalyzes the initial and rate-regulated first-stage pathway of glycerol lipid metabolism, directing acetyl-CoA towards triacylglycerol synthesis and away from degradation pathways in animal lipid metabolism pathways [31]. In addition, this study observed that *BDH1* inhibited TAG synthesis in GMECs, possibly due to the synergistic role of *BDH1* and *GPAM* genes in this process.

**Fig. 7:**
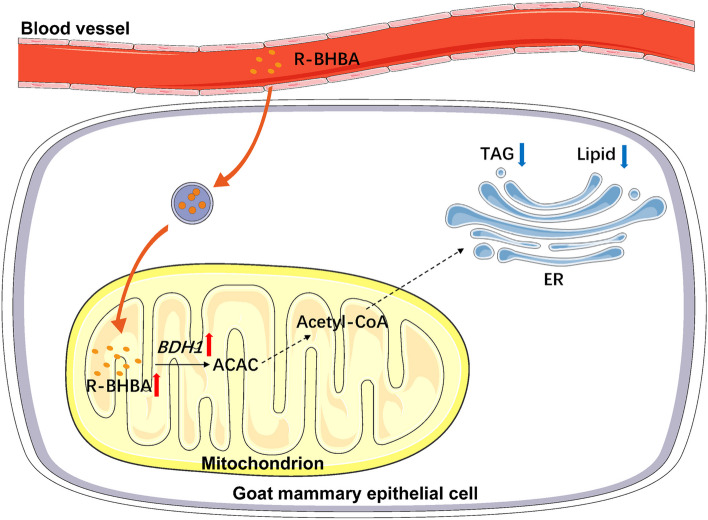
A model summarizing the effect of *BDH1* on lipid metabolism in GMECs (partly created by Servier Medical Art)

In lipid metabolism, *BDH1* catalyzes the conversion of BHBA to ACAC, which is further transformed into acetyl-CoA [32]. This study observed a significant increase in the relative mRNA expression level of *BDH1* with an elevated BHBA concentration, indicating a positive correlation between BHBA and *BDH1* in regulating lipid metabolism in mammary gland of dairy goats. Co-treatment of siRNA-BDH1 with BHBA resulted in a significant increase in intracellular TAG content, suggesting that interfering with *BDH1* could counteract the inhibitory effect of BHBA on lipid metabolism. These findings align with the known role of BHBA in accelerating lipid metabolism and inhibiting glycolysis-activated oxidative phosphorylation [33]. However, contrasting results from a study on subclinical ketosis in dairy cows indicated that BHBA increased cellular TAG content [34]. This inconsistency may stem from the different molecular conformations of D-L-β-hydroxybutyric acid and (R)-β-hydroxybutyric acid, both ketone bodies and substrates for *BDH1* in this experiment. Studies have demonstrated that feeding lambs concentrates significantly elevates rumen and serum BHBA levels and alters the cellular composition of rumen epithelial cells [35]. BHBA activates the NOTCH signaling pathway and inhibits the proliferation of rumen epithelial cells by suppressing BDH1 expression, which further inhibits HDAC activity [36]. We hypothesized that modifying the feeding practices of dairy goats to regulate BHBA levels could improve goat milk quality. Overall, a schematic summary of the effect of *BDH1* on lipid metabolism in GMECs is shown in Fig. 7.

## Conclusion

In summary, the CDS region of the *BDH1* gene in dairy goats was successfully cloned, with a length of 1,035 bp. *BDH1* serves as a negative regulator of lipid metabolism in GMECs, and lipid metabolism-related gene expression is upregulated by interfering with *BDH1*. Furthermore, supplementation of R-BHBA similarly negatively regulated lipid metabolism, and *BDH1* counteracts the inhibitory effect of R-BHBA on lipid metabolism in mammary epithelial cells of dairy goats. The results are expected to offer a novel theoretical foundation for enhancing milk quality in dairy goats. Future studies could further elucidate the mechanism through which the BDH1 gene regulates lipid metabolism in mammary gland.

## Supplementary Information

Supplementary Material 1: Supplementary Table S1. Online software information. Supplementary Table S2. Cloning of goat *BDH1* gene and designing of restriction primer. Supplementary Table S3. Design of *BDH1*-specific siRNA sequences online. Supplementary Table S4. Primer pairs for RT-qPCR analysis. Supplementary Table S5. Antibodies information. Supplementary Table S6. *BDH1* in the mammary gland of transcriptome sequences. Supplementary Figure S1. Volcano plot from mammary tissues of dairy goats with low and high milk fat percentages. Supplementary Figure S2. CDS region coding sequence and corresponding amino acid sequence of *BDH1*. Supplementary Figure S3. The predicted phosphorylation sites of *BDH1*. Supplementary Figure S4. The transmembrane structure prediction of *BDH1*.

## Data Availability

None of the data were deposited in an official repository. The datasets used or analyzed during the present study are available from the corresponding author on reasonable request. The transcriptome data have not been published.

## Abbreviations

GMECs: Mammary epithelial cells of dairy goats
ACAC: Acetoacetic acid
BHBA: β-Hydroxybutyric acid
RT-qPCR: Quantitative real time PCR
BSA: Bovine serum albumin
CCK-8: Cell counting kit-8
TAG: Triacylglycerol
CDS: Coding sequence
cDNA: Complementary DNA
siRNA: Small interfering RNA
*BDH1*: 3-Hydroxybutyrate dehydrogenase 1
*SCD*: Stearoyl-CoA desaturase
*SREB**F**1*: Sterol regulatory element binding transcription factor 1
*NR1H3*: Nuclear receptor subfamily 1 group H member 3
*FASN*: Fatty acid synthase
*ACACA*: Acetyl-CoA carboxylase alpha
*ELOVL6*: ELOVL fatty acid elongase 6
*LPL*: Lipoprotein lipase
*FABP3*: Fatty acid binding protein 3
*ACSL1*: Acyl-CoA synthetase long chain family member 1
*CD36*: *CD36* Molecule
*GPAM*: Glycerol-3-phosphate acyltransferase, mitochondrial
*DGAT1*: Diacylglycerol O-acyltransferase 1
*ATGL*: Patatin like phospholipase domain containing 2
*XDH*: Xanthine dehydrogenase
*PLIN1*: Perilipin 1
*RPS9*: Ribosomal protein S9
*UXT*: Ubiquitously expressed prefoldin like chaperone
