# Supplementary material for: BDH1 identified by transcriptome has a negative effect on lipid metabolism in mammary epithelial cells of dairy goats
Source: BMC Genomics. 2025 Jan 24;26:66. doi: 10.1186/s12864-025-11245-1 (PMC11761236; doi:10.1186/s12864-025-11245-1)
Supplement: Supplementary file 1 — Supplementary Material 1: Supplementary Table S1. Online software information. Supplementary Table S2. Cloning of goat BDH1 gene and designing of restriction primer. Supplementary Table S3. Design of BDH1-specific siRNA sequences online. Supplementary Table S4. Primer pairs for RT-qPCR analysis. Supplementary Table S5. Antibodies information. Supplementary Table S6. BDH1 in the mammary gland of transcriptome sequences. Supplementary Figure S1. Volcano plot from mammary tissues of dairy goats with low and high milk fat percentages. Supplementary Figure S2. CDS region coding sequence and corresponding amino acid sequence of BDH1. Supplementary Figure S3. The predicted phosphorylation sites of BDH1. Supplementary Figure S4. The transmembrane structure prediction of BDH1. [file 12864_2025_11245_MOESM1_ESM.docx]

Table S1 Online software information.

| Software | Version | Website |
| --- | --- | --- |
| Phyre2 | 2.2 | https://www.sbg.bio.ic.ac.uk/phyre2/ |
| NetPhosK | 3.1 | https://services.healthtech.dtu.dk/services/NetPhos-3.1/ |
| TMHMM | 2.0 | https://services.healthtech.dtu.dk/services/TMHMM-2.0/ |

Table S2 Cloning of goat *BDH1* gene and designing of restriction primer.

| Gene | Accession number | Sequence（5' to 3'） | Tm/℃ | bp^3^ |
| --- | --- | --- | --- | --- |
| BDH1^1^ | XM_018046468.1 | F:GAGCCTGACCATTCTGCCTT | 60 | 1218 |
|  |  | R:TCTAGTTAATGCTCAGACTCGTGG |  |  |
| BDH1^2^ | XM_018046468.1 | F:***CCC*AAGCTT**ATGCTGACAGCCCGCCT | 60 | 1035 |
|  |  | R:***GC*TCTAGA**TCAGTGGATATAGATCCTG |  |  |

Italics are protected bases, and underlines are enzyme cleavage sites.

*BDH1*, 3-hydroxybutyrate dehydrogenase 1.

^1^ Clone primers

^2^ Primers digested by enzyme

^3^ Amplicon size in base pair (bp)

Table S3 Design of *BDH1*-specific siRNA sequences online.

| Name | Sequence | |
| --- | --- | --- |
|  | Sense (5'-3') | Antisense (5'-3') |
| siRNA-NC | UUCUCCGAACGUGUCACGUTT | ACGUGACACGUUCGGAGAATT |
| siRNA-608 | GACCCUGAGAAAGGCUUAUTT | AUAAGCCUUUCUCAGGGUCTT |
| siRNA-424 | GGCCAAACAUCUGCAUUCATT | UGAAUGCAGAUGUUUGGCCTT |
| siRNA-1032 | GCAGGAAGUACUUCGAUGATT | UCAUCGAAGUACUUCCUGCTT |

Table S4 Primer pairs for RT-qPCR analysis.

| Gene | Primer^1^ | Sequence (5′ to 3′) | bp^2^ | Tm (℃) |
| --- | --- | --- | --- | --- |
| *BDH1* | F | AGGCTCGCTGCTGTTTAACT | 201 | 60 |
|  | R | CCATCACTCCCTTTGTCCTTCA |  |  |
| *SCD* | F | CCATCGCCTGTGGAGTCAC | 256 | 60 |
|  | R | GTCGGATAAATCTAGCGTAGCA |  |  |
| *SREBF1* | F | CTGCTGACCGACATAGAAGACAT | 81 | 60 |
|  | R | GTAGGGCGGGTCAAACAGG |  |  |
| *NR1H3* | F | CATCAACCCCATCTTCGAGTT | 163 | 60 |
|  | R | CAGGGCCTCCACATATGTGT |  |  |
| *FASN* | F | GGGCTCCACCACCGTGTTCCA | 226 | 60 |
|  | R | GCTCTGCTGGGCCTGCAGCTG |  |  |
| *ACACA* | F | CTCCAACCTCAACCACTACGG | 171 | 60 |
|  | R | GGGGAATCACAGAAGCAGCC |  |  |
| *ELOVL6* | F | GGAAGCCTTTAGTGCTCTGGTC | 205 | 60 |
|  | R | ATTGTATCTCCTAGTTCGGGTGC |  |  |
| *LPL* | F | CATCAACCCCATCTTCGAGTT | 163 | 60 |
|  | R | CAGGGCCTCCACATATGTGT |  |  |
| *FABP3* | F | GATGAGACCACGGCAGATG | 120 | 60 |
|  | R | GTCAACTATTTCCCGCACAAG |  |  |
| *ACSL1* | F | GTGGGCTCCTTTGAAGAACTGT | 120 | 60 |
|  | R | ATAGATGCCTTTGACCTGTTCAAAT |  |  |
| *CD36* | F | GTACAGATGCAGCCTCATTTCC | 81 | 60 |
|  | R | TGGACCTGCAAATATCAGAGGA |  |  |
| *GPAM* | F | ATTGACCCTTGGCACGATAG | 188 | 60 |
|  | R | AACAGCACCTTCCCACAAAG |  |  |
| *DGAT1* | F | CCACTGGGACCTGAGGTGTC | 101 | 60 |
|  | R | GCATCACCACACACCAATTCA |  |  |
| *ATGL* | F | GGAGCTTATCCAGGCCAATG | 180 | 60 |
|  | R | TGCGGGCAGATGTCACTCT |  |  |
| *XDH* | F | GATCATCCACTTTTCTGCCAATG | 100 | 60 |
|  | R | CCTCGTCTTGGTGCTTCCAA |  |  |
| *PLIN1* | F | GATGAGACCACGGCAGATG | 120 | 60 |
|  | R | GTCAACTATTTCCCGCACAAG |  |  |
| *RPS9* | F | CCTCGACCAAGAGCTGAAG | 64 | 60 |
|  | R | CCTCCAGACCTCACGTTTGTTC |  |  |
| *UXT* | F | TGTGGCCCTTGGATATGGTT | 101 | 60 |
|  | R | GGTTGTCGCTGAGCTCTGTG |  |  |

^1^ Primer direction (F-forward, R-reverse) and hybridization position on the sequence.

^2^ Amplicon size in base pair (bp)

Table S5 Antibodies information.

| Name | Article number | Antibody concentration | Manufacturers |
| --- | --- | --- | --- |
| BDH1 | 67448-1-Ig | 1:2000 | Proteintech |
| β-Tublin | 10094-1-AP | 1:2000 | Proteintech |
| Goat anti rabbit IgG | SA00001-1 | 1:10000 | Proteintech |

Table S6 *BDH1* in the mammary gland of transcriptome sequences.

| Gene | High_normalize | Low_normalize | FoldChange | pval |
| --- | --- | --- | --- | --- |
| *BDH1* | 2560.015882 | 4117.252128 | 0.621777779 | -0.685529037 |


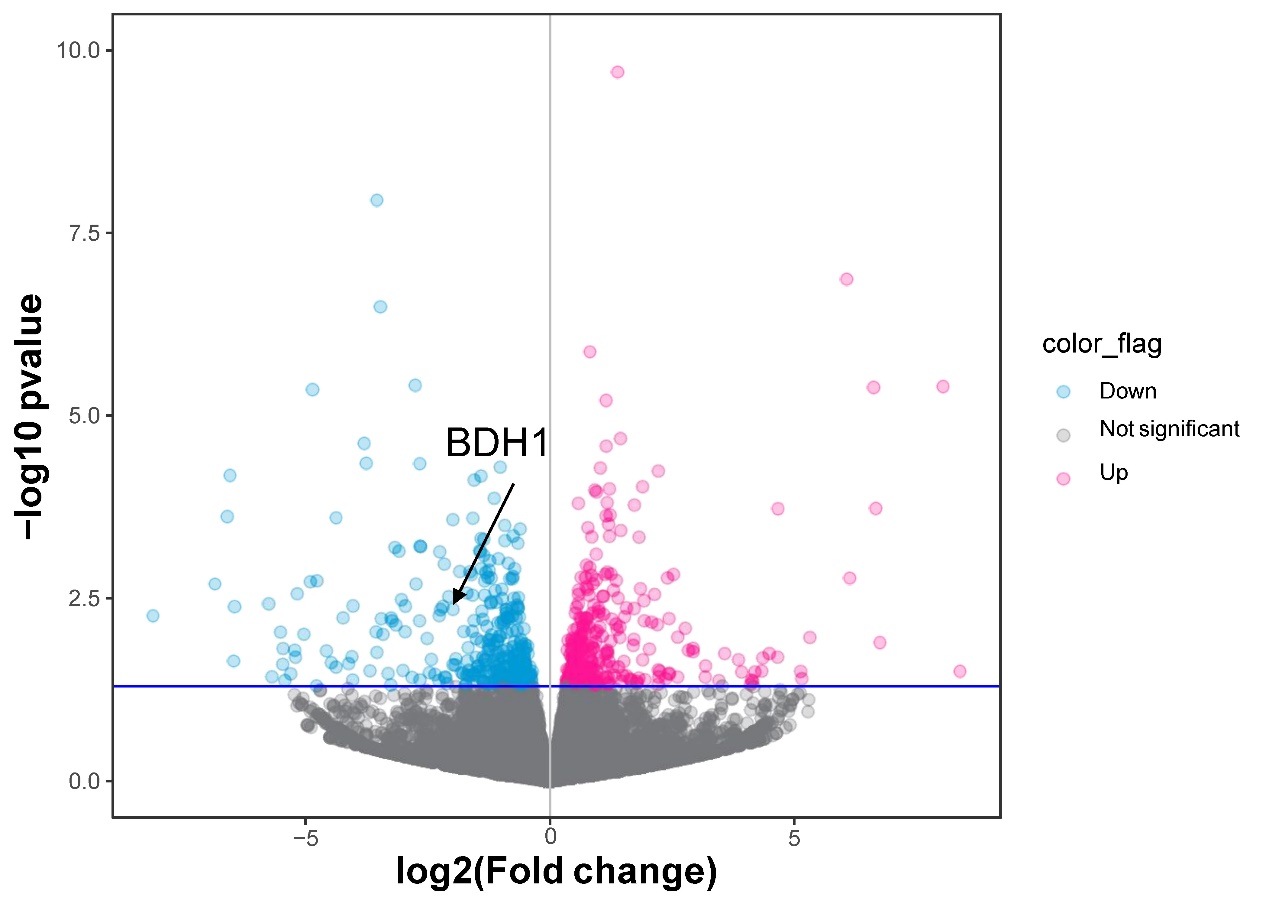


**Figure S1**. Volcano plot from mammary tissues of dairy goats with low and high milk fat percentages.


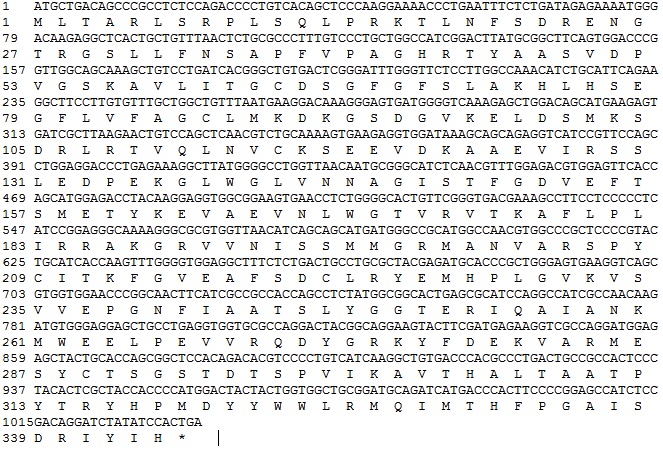


**Figure S2**. CDS region coding sequence and corresponding amino acid sequence of *BDH1*.


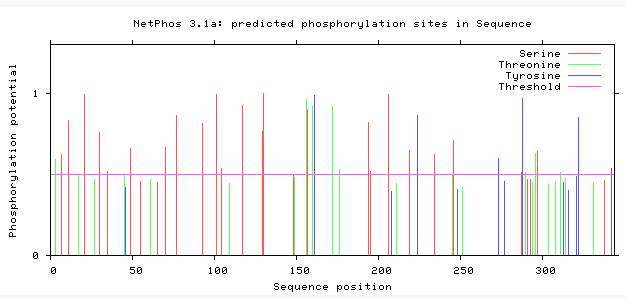


**Figure S3.** The predicted phosphorylation sites of *BDH1*.


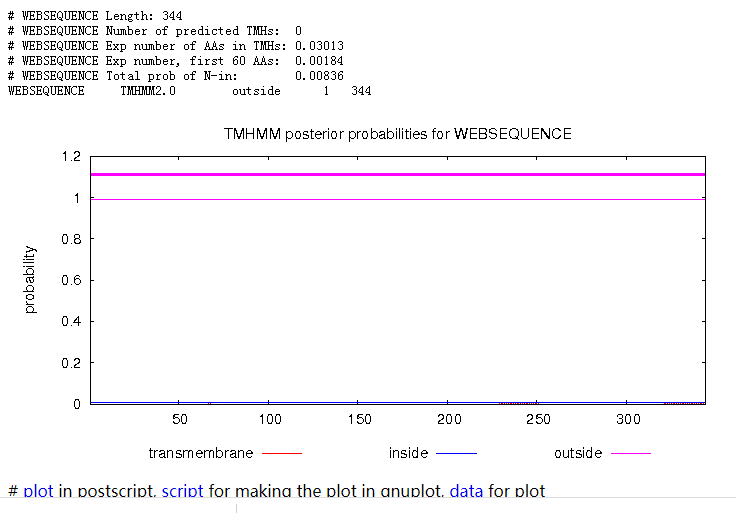


**Figure S4.** The transmembrane structure prediction of *BDH1*.
